# Effects of salivary amylase gene copy number on metabolic health

**DOI:** 10.3389/fnut.2026.1815044

**Published:** 2026-08-28

**Authors:** Sri Lakshmi Sravani Devarakonda, Kalem D. Hanlon, Alex P. Loinard-González, Bethany P. Cummings, Angela C. Poole

**Affiliations:** 1 Division of Nutritional Sciences, Cornell University, Ithaca, NY, United States; 2 Department of Surgery, School of Medicine, Center for Alimentary and Metabolic Science, University of California, Davis, Davis, CA, United States; 3 Department of Molecular Biosciences, School of Veterinary Medicine, University of California, Davis, Davis, CA, United States

**Keywords:** *AMY1*, amylase, diabetes, glucose, insulin resistance, obesity, precision nutrition, starch

## Abstract

Precision nutrition is the personalization of dietary recommendations based on characteristics such as genetics, the microbiome, lifestyle, environment, and baseline metabolic state. One potential basis for the development of guidelines is the characterization of gene-diet interactions. In this narrative review, we evaluate the published literature reporting associations between salivary amylase gene copy number and metabolic health. The salivary amylase enzyme facilitates starch digestion and is encoded by *AMY1*, a gene copy number (CN) variant. Humans have 2–20 copies, and *AMY1* CN has been associated with metabolic health conditions such as obesity and insulin resistance. Studies of these associations have conflicting findings. The objectives of this review are to assess the findings from studies testing associations between *AMY1* CN and adiposity, glucose metabolism, and gut microbiome composition; to explore possible mechanisms underlying the effects on metabolic health; and to identify knowledge gaps requiring additional research. To identify relevant articles, we searched PubMed, Web of Science, Cumulative Index to Nursing and Allied Health Literature, and Centre for Agricultural and Biosciences International for articles focused on *AMY1* CN and one or more of the following: body mass index, glucose metabolism, and the microbiome. Key findings are that *AMY1* CN has a positive association with postprandial glucose response and that a high *AMY1* CN is protective against insulin resistance. *AMY1* CN alone does not appear to be an accurate predictor of adiposity, and the relationship is likely convoluted by habitual starch intake, genetic background, and lifestyle factors. Future studies are required to determine how *AMY1* CN could be used as a biomarker or to inform precision nutrition protocols to achieve metabolic health outcomes.

## 1 Introduction

Despite therapeutic advancements, the incidence of metabolic disorders, such as obesity and type 2 diabetes, has progressively increased in adults and is a major financial burden on healthcare systems. The prevalence of obesity has more than doubled since 1990, with more than 1 billion people experiencing obesity worldwide (1). In the U.S. alone, obesity incurs over 1.73 trillion dollars in cost annually, equating to almost 10% of GDP (2). The prevalence of type 2 diabetes (T2D) has increased concurrently with the international diabetes federation estimating that 1 in 8 people will have diabetes by 2045, an increase of 46% (3). T2D costs the U.S. healthcare system $327 billion in 2017 (4).

Although there are effective pharmaceutical treatments for T2D and obesity, including metformin and glucagon-like peptide-1 receptor agonists (GLP-1 RAs), there are numerous benefits to preventing or reversing these conditions with lifestyle interventions, including dietary changes. Metformin is widely used and well-tolerated by most patients, but gastrointestinal issues are the most frequently reported side effects, affecting up to 21% of patients (5–7). In some patients, GLP-1 RAs cause gastrointestinal side effects (nausea, vomiting, diarrhea), especially at higher doses (8–12). GLP-1 RAs are expensive and often not fully covered by insurance, creating significant access barriers, which is one reason why some eligible patients do not receive these drugs despite guideline recommendations (13–15). Some patients may turn to compounding pharmacies if commercial products are unaffordable or unavailable due to episodic shortages, which poses concerns about safety and quality (16–19). Additionally, the use of metformin or GLP-1 RAs to manage diabetes or weight should be thought of by patients as a life-long intervention, with cessation of GLP-1 RAs frequently causing patients to regain weight and metabolic markers to return to previous values (20, 21). A portion of the weight lost using GLP-1 RAs is lean mass (22); GLP1-RAs are commonly associated with micronutrient deficiencies (23); and there is a limited amount of data regarding the effects of long-term use. Furthermore, no single medication can confer all the benefits of appropriate dietary modification for improving body composition, glucose metabolism, cardiovascular health, and cancer risk.

Most patients with prediabetes or obesity prefer lifestyle interventions over medication, citing concerns about drug side effects and dependency (24–28). However, broad population-based guidelines regarding lifestyle and dietary modifications have had limited success, and personalized approaches, in which dietary recommendations are tailored to the person, are an area of active study. Precision nutrition aims to utilize individual attributes such as health- and diet-related genotypes and gut microbiome composition to tailor dietary advice. Genetic heritability partly accounts for the phenotypic variation in body mass index (40–70%) (29, 30) and glucose metabolism (~30–40%) (31–33). Delineating the mechanisms underlying the interactions between host genotype and dietary intake to influence metabolic health would contribute to the development of precision nutrition protocols. A candidate enzyme involved in starch digestion and previously associated with BMI and glucose metabolism is encoded by the gene copy number variant *AMY1*.

Dozens of studies have tested associations between salivary amylase gene copy number and metabolic health parameters (Tables 1–3; Supplementary Tables S2–S4). Amylase is an enzyme that helps mammals digest dietary starch. Salivary amylase is encoded by a gene copy number variant, *AMY1*, which has an instructive evolutionary history. Carbohydrate intake influences body weight and blood glucose, so variations in amylase enzyme dosage could affect these outcomes. Variability in the ratio of digestible carbohydrates to dietary fiber that reaches the gut microbiome could further impact metabolic health through the activity of microbial metabolites. In this review, we summarize the physiology, genomic arrangement, and evolutionary history of amylase. Then we discuss reported associations between salivary amylase gene copy number and metabolic health, including studies reporting interactions with starch intake. This review contributes to the foundation for future research directing precision nutrition strategies.

**Table 1:** Summary of studies reporting associations between *AMY1* CN and adiposity.

Author	Year	*AMY1* CN grouping designation	Size of cohort	Ethnicity	Association with *AMY1* CN
Al-Akl et al. (98)	2020	High CN ≥ 7 vs. Low CN < 7	929	Qatari	Fat mass index and body adiposity index are higher in men with low *AMY1* CN.
Al-Akl et al. (98)	2022	Continuous variable	1,499	Qatari	No association with adiposity or glycemic markers; mean and median *AMY1* CN did not differ between normal versus overweight or obese; mean and median *AMY1* CN did not differ between normoglycemic versus diabetes.
Atkinson et al. (78)	2018	Study 1: Continuous variable	Study 1: 201	Study 1: European Caucasian and Asian	Study 1: No association with weight, BMI, fasting glucose, HOMA-IR, QUICKI, glucose tolerance, nor Matsuda ISI.
Barber et al. (79)	2020	ProFiMet Study: Continuous variable	ProFiMet Study: 80	ProFiMet Study: Not specified	ProFiMet Study: Inverse correlation with total visceral fat volume and visceral fat volume within the umbilical area. Direct correlation with serum adiponectin.
Bonnefond et al. (72)	2017	Continuous variable	4,834	European (French)	Nominal negative association with BMI.
Christensen et al. (137)	2022	Study 1: High CN > 6.8 vs. Low CN < 6.8; Study 2: High CN > 6.4 vs. Low CN < 6.4	Study 1: 34; Study 2: 36	European (Danish)	Low *AMY1* CN associated with body fat loss and a high baseline *Prevotella* abundance on a whole grain diet; no association in high AMY1 CN individuals.
Falchi et al. (66)	2014	Continuous variable	6,290	European; Asian	Inverse association with obesity and BMI.
Farrell et al. (81)	2021	Study 1: Continuous variable	Study 1: 1,764	European (Swedish)	Study 1: Inverse association with fasting glucose and BMI at high starch intake; positive association at low starch intake.
Grammatikopoulou et al. (144)	2020	Continuous variable	43	Caucasian	No association with BMI, odd diploid CN was associated with greater body weight, fat mass, waist, and hip circumferences compared to even diploid CN.
Hasegawa et al. (99)	2022	Continuous variable	676 for BMI and obesity; 587 for HbA1c and diabetes	Northern Japanese	Positive association of *AMY1* and *AMY2* with diabetes, obesity, HbA1c and BMI.
Hjorth et al. (135)	2020	High CN > 6.5, Low CN < 6.5	62	European (Danish)	Baseline *Prevotella*/*Bacteroides* ratio predicted greater weight loss with a high fiber diet in low *AMY1* individuals, no association in high *AMY1* individuals.
León-Mimila et al. (123)	2018	High CN ≥ 10 vs. Low CN ≤ 4	920 (obesity); 45 (microbiota)	Mexican	Inverse association with obesity, positive correlation with *Prevotella* abundance
Marquina et al. (145)	2019	High CN > 4 vs. Low CN ≤ 4	57	Caucasian (28%), South/Central Asian (39%), Northeast/Southeast Asian (23%) and other (6%)	Inverse association with fat mass, LDL, and inflammatory markers, whereas no association was found with glycemic measures.
Mayneris-Perxachs et al. (146)	2020	High CN > 4 vs. Low CN ≤ 4	57	Caucasian (28%), South/Central Asian (39%), Northeast/Southeast Asian (23%) and other (6%)	Inverse association with BMI and fat mass, LDL, and inflammatory markers, while no association was found for glycemic measures (glucose, insulin, and insulin sensitivity).
Nayema et al. (147)	2023	Continuous variable	1,150	Japanese	No association with BMI.
Pinho et al. (148)	2018	CN ≤ 5, 5 < CN ≤ 7, CN 7 < CN ≤ 9, CN ≥ 10	262	European (Portuguese)	Inverse association with BMI with CN ≥ 10.
Rossi et al. (149)	2021	Continuous variable	2,935	Qatari (Bedouin Arabs, Persian, East African, Admixed)	Inversely associated with total and trunk fat in Arabs but not Persians, and negatively associated with dietary restraint following weight gain.
Rukh et al. (150)	2017	Continuous variable	4,800	European (Swedish)	Significant effect of interaction with starch intake on BMI and body fat percentage; BMI tended to be negatively associated with *AMY1* CN in the low starch group, and positively associated with *AMY1* CN in the high starch group.
Shwan and Armour (151)	2019	Continuous variable	4,235	European (English)	No association with BMI.
Tayhan et al. (155)	2025	Continuous variable	85 (women only)	Turkish	Inverse association with body weight, BMI, fat percentage, and waist circumference.
Usher et al. (60)	2015	Continuous variable	4,464	Estonian, other European	No association with BMI.
Valsesia et al. (96)	2019	Continuous variable	761	European, multi-center	Modest negative association with BMI; no association with glycemic parameters or weight loss outcomes. No effects involving interaction with starch intake.
Viljakainen et al. (152)	2015	Continuous variable	132	Finnish	Inverse association with BMI and fat percentage in females with obesity when comparing CN of individuals with and without obesity for each sex.
Yong et al. (153)	2016	Continuous variable	1,077	Chinese and Malay	No association with BMI or obesity.
Zhan et al. (80)	2021	Continuous variable	560	Chinese	Negative correlation with BMI and triglycerides. CN lower in patients with metabolic syndrome in the <45 age group, but no correlation in the ≥45 age group.

See Supplementary Table S2 for more details. CN = copy number; BMI = body mass index; HOMA-IR = homeostatic model assessment of insulin resistance; QUICKI = Quantitative Insulin Sensitivity Check Index; HbA1c = hemoglobin A1c.

**Table 2:** Summary of studies reporting associations between *AMY1* CN and glucose homeostasis.

Author	Year	*AMY1* CN grouping designation	Size of cohort	Ethnicity	Association with *AMY1* CN
Al-Akl et al. (98)	2021	Continuous variable	929	Qatari	No association with diabetes.
Al-Akl et al. (98)	2022	Continuous variable	1,499	Qatari	No association with adiposity or glycemic markers (HOMA-IR, HOMA-β); mean and median *AMY1* CN did not differ between normal versus overweight or obese; mean and median *AMY1* CN did not differ between normoglycemic versus diabetes.
Alberti et al. (84)	2015	Continuous variable	10	South American	No significant association with postprandial glycemia or insulin response.
Atkinson et al. (78)	2018	Study 1: Continuous variable; Study 2: Continuous variable; Study 3: High: mean AMY1 CN = 10.4, Low: mean *AMY1* CN = 3.1; Study 4: High CN ≥ 7 vs. Low CN ≤ 5	Study 1: 201; Study 2: 114; Study 3: 40; Study 4: 30	Study 1: European Caucasian and Asian; Study 2: European Caucasian and Asian; Study 3: European Caucasian; Study 4: Not specified	Study 1: No association with weight, BMI, fasting glucose, HOMA-IR, QUICKI, glucose tolerance, nor Matsuda ISI; Study 2: Positive correlation with glycemic response to 6 starchy foods, but not canned fruit or sucrose; Study 3: Positive association with glycemic response to white bread and pasta, but not insulin; Study 4: Inverse association with methane gas production following starch intake, but not hydrogen.
Barber et al. (79)	2020	ProFiMet Study: Continuous variable; OSC (Oral Starch Challenge) Study: High CN = 9–12 vs. Low CN = 4–6	ProFiMet Study: 80; OSC Study: 17	ProFiMet Study: Not specified; OSC Study: White (Irish/British)	ProFiMet Study: Direct correlation with (OGTT-derived) Oral Glucose Insulin Sensitivity score. Inverse association with total visceral fat volume. No significant association with whole body IR, HOMA-IR, insulin sensitivity index; OSC Study: Inverse association with fasting blood glucose. Change in blood glucose (fasting to 30 min) in response to starch is higher in the high *AMY1* group than in the low group.
Choi et al. (94)	2015	Continuous variable	1,257	Korean	Negative correlation with HOMA-IR.
Farrell et al. (81)	2021	Study 1: Continuous variable; Study 2: High CN ≥ 10 vs. Low CN ≤ 4	Study 1: 1,764; Study 2: 19	European (Swedish)	Study 1: Inverse association with fasting glucose and BMI at high starch intake, positive association at low starch intake; Study 2: Positive association with PPGR and insulinemia following 40 g of starch, with a smaller and non-significant difference following 80 g of starch, respectively.
Hamid et al. (95)	2021	CN groups: 1–4, 5–6, 7–9, 10 and above	3,624	European (Swedish)	Inverse association with HOMA-IR and insulin with high starch intake, stronger association in CN > 10, no association without accounting for starch intake.
Hasegawa et al. (99)	2022	Continuous variable	676 for BMI and obesity; 587 for HbA1c and diabetes	Northern Japanese	Positive association of *AMY1* and *AMY2* with diabetes, obesity, HbA1c, and BMI.
Higuchi et al. (85)	2020	Continuous variable; High CN 8–14 vs. Low CN 4–7	60	Japanese	*AMY1* inversely correlated with HbA1c; low *AMY1* CN significantly associated with an HbA1c of ≥ 5.4% compared to high *AMY1* CN.
Liu et al. (93)	2020	BPRHS Study: High CN ≥ 6 vs. Low CN < 6; GOLDN Study: High CN ≥ 5 vs. Low CN < 5	BPRHS Study: 749; GOLDN Study: 980	BPRHS Study: Puerto Rican; GOLDN Study: European	People with low CN tend to have increased insulin resistance as they age.
Lv et al. (97)	2023	Continuous variable	60	Chinese	No association with T2D or T2D with hypothyroidism
Mandel and Breslin (82)	2012	High CN 4–11 vs. Low CN 2–4	14	Mixed	Inverse association with PPGR; positive association with preabsorptive insulin response.
Marquina et al. (145)	2019	High CN > 4 vs. Low CN ≤ 4	57	Caucasian (28%), South/Central Asian (39%), Northeast/Southeast Asian (23%) and other (6%)	Inverse association with fat mass, LDL, and inflammatory markers; no association was found with glycemic measures (insulin sensitivity or secretion).
Mayneris-Perxachs et al. (146)	2020	Low CN ≤ 4 vs. High CN > 4	57	Caucasian (28%), South/Central Asian (39%), Northeast/Southeast Asian (23%) and other (6%)	Inverse association with BMI and fat mass, LDL, and inflammatory markers; no association was found for glycemic measures (glucose, insulin, and insulin sensitivity).
Tan et al. (83)	2016	Continuous variable	75	Malay, Chinese, Asian-Indian	No association with postprandial glycemia.
Valsesia et al. (96)	2019	Continuous variable	761	European, multi-center	Modest negative association with BMI; no association with glycemic parameters (fasting plasma glucose, HOMA-IR, Matsuda index, OGTT) nor weight loss outcomes; no interaction effect with starch intake on outcome variables.
Zhan et al. (154)	2021	Continuous variable	560	Chinese	Inverse association with BMI and triglycerides, CN is lower in MetS patients compared to healthy controls in the <45 age group, but no correlation in the >45 group.
Zhang et al. (80)	2023	High CN ≥ 6 vs. Low CN ≤ 2	16	Chinese	Inverse association with fasting insulin and HOMA-IR, lower postprandial blood glucose and insulin following starch intake for the low CN group.

See Supplementary Table S3 for more details. CN = copy number; BMI = body mass index; HOMA-IR = homeostatic model assessment of insulin resistance; QUICKI = Quantitative Insulin Sensitivity Check Index; HbA1c = hemoglobin A1c.

**Table 3:** Summary of studies reporting associations between *AMY1* CN and the microbiome.

Author	Year	*AMY1* CN grouping designation	Size of Cohort	Ethnicity	Association with *AMY1* CN
Barber et al. (79)	2020	ProFiMet Study: Continuous variable; OSC (Oral Starch Challenge) Study: High CN = 9–12 vs. Low CN = 4–6	ProFiMet Study: 80; OSC Study: 17	ProFiMet Study: Not specified; OSC Study: White (Irish/British)	No correlation with any measure of microbiota.
Christensen et al. (137)	2022	Study 1: High CN > 6.8 vs. Low CN < 6.8; Study 2: High CN > 6.4 vs. Low CN < 6.4	Study 1: 34; Study 2: 36	European (Danish)	Baseline *Prevotella* abundance positively correlated with fat loss on a whole grain diet only in individuals with a low *AMY1* CN. Baseline *Prevotella* was not correlated with AMY1 CN.
Devarakonda et al. (139)	2024	Continuous variable	59	Mixed	Mean SAA was positively associated with the abundance of *Sutterella* at the end of the digestible starch treatment.
Farrell et al. (81)	2021	Study 1: Continuous variable	Study 1: 1,412	European (Swedish)	No correlation with 64 genera assessed. Nominally significant interaction between habitual starch intake and *AMY1* CN with 2 out of 64 genera (*Megasphaera and Acidaminococcus*).
Hasegawa et al. (99)	2022	Continuous variable	16S rRNA: 1,787; shotgun metagenomics: 617	Northern Japanese	*Dialister* increased in males and decreased in females with increasing *AMY1* CN. Gene abundance analysis revealed a negative association with *AMY1* CN and the acarbose and validamycin biosynthesis pathways.
Hjorth et al. (135)	2020	High CN > 6.5, Low CN < 6.5	62	European (Danish)	In low *AMY1* CN individuals, the baseline *Prevotella* to *Bacteroides* ratio predicted higher weight loss on a high fiber diet compared with the average Danish diet. Baseline *Prevotella* was not correlated with *AMY1* CN, although all 0-*prevotella* individuals had high CNs.
León-Mimila et al. (123)	2018	High CN ≥ 10 vs. Low CN ≤ 4	920 (obesity); 45 (microbiota)	Mexican	*Prevotella* abundance was significantly positively associated with *AMY1* CN in adults. *P. copri* was 2 × higher in adults with CN ≥ 10 compared to those with CN ≤ 4.
Poole et al. (122)*	2019	High CN > 8 (*AMY1H*) vs. Low CN < 5 (*AMY1L*)	20	Mixed	Relative abundances of Ruminococcaceae OTUs were greater in the *AMY1H* group. Most *Bacteroides* are enriched in *AMY1H* individuals. *Prevotella copri* was enriched in *AMY1H* at one time point. Enrichment of CAZymes classes in *AMY1L* individuals. FMT from *AMY1H* human donors associated with greater adiposity in germfree mouse recipients than in germfree mice with FMT from *AMY1L* human donors.

See Supplementary Table S4 for more details. FMT = fecal microbiota transplant; OTU = operational taxonomic unit.

### 1.1 Amylase physiology

Amylase was among the first enzymes discovered and is produced by microbes, plants, and animals (34, 35). Amylase breaks down starch and glycogen into simpler sugars, and there are three forms—alpha-amylase, beta-amylase, and gamma-amylase—which target different bonds in a substrate (36). The amylase genes encoded in the human genome produce alpha-amylases, which are the subject of this review. Salivary glands secrete amylase in the mouth, and the pancreas secretes amylase into the small intestine to help digest dietary starches (37). Amylase is the most abundant enzyme in human saliva (38). Other organs contain amylase, but at orders of magnitude lower than the salivary glands and pancreas (39). Serum amylase is derived primarily from the pancreas and salivary glands and is used to diagnose pancreatitis (40, 41). This review focuses on salivary amylase, which is more frequently measured in research studies than serum amylase.

**Figure 1:**
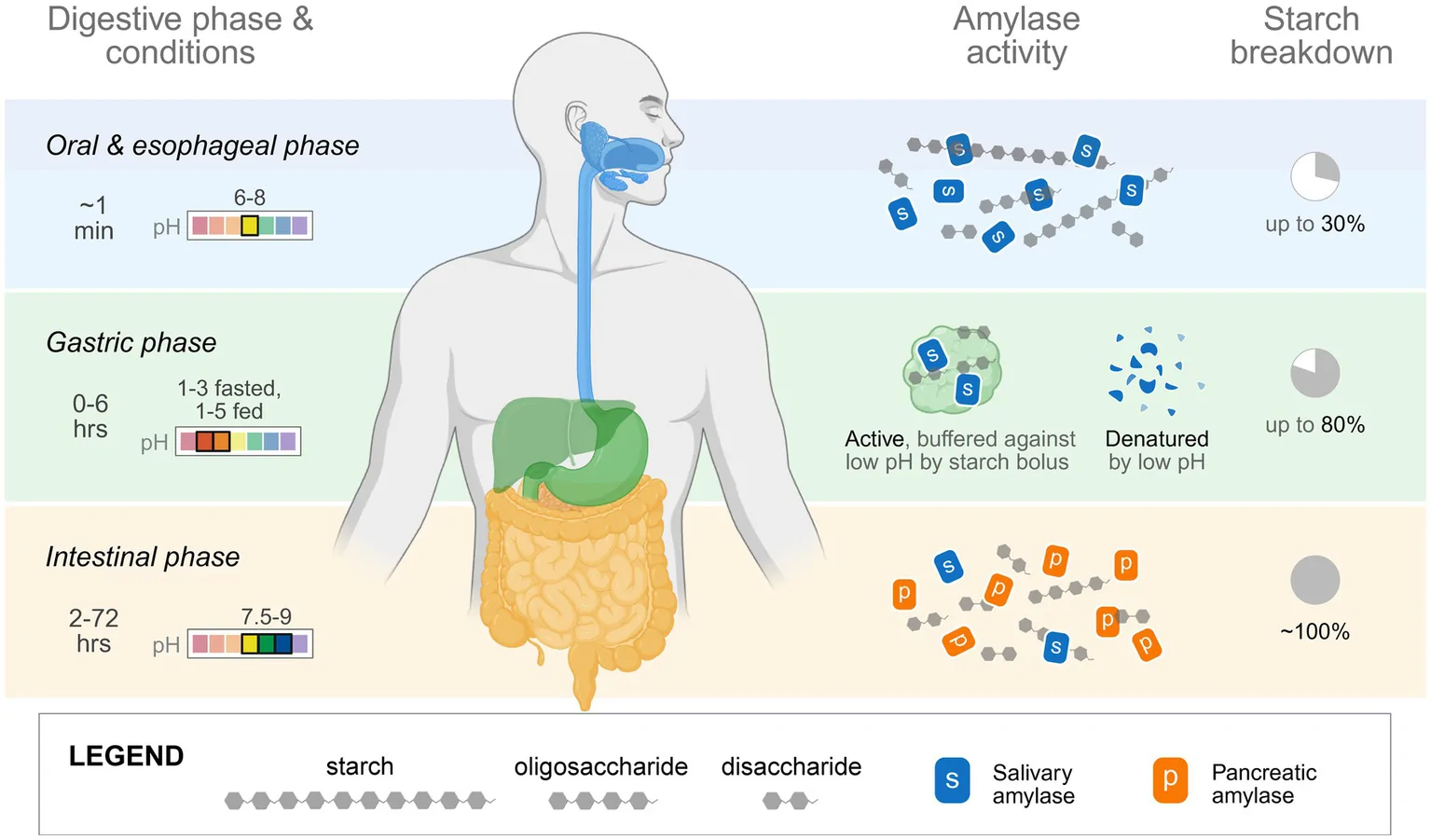
Spatiotemporal expression and activity of amylase throughout the gastrointestinal tract. Salivary amylase is secreted in the oral cavity to begin starch degradation during the oral and esophageal phase. During the gastric phase, the stomach’s acidic pH is buffered by the food bolus, maintaining partial enzymatic activity of salivary amylase throughout. Pancreatic amylase is secreted into the intestinal lumen following gastric emptying to complete starch breakdown to disaccharides with the remaining active salivary amylase. Created in BioRender. Poole, A. (https://BioRender.com/16im9jj) is licensed under CC BY 4.0.

Salivary and pancreatic amylase are encoded by two different genes, *AMY1* and *AMY2*, which have variable copy number in humans. The *AMY1* gene encodes the salivary amylase enzyme. The number of gene copies is positively correlated with salivary amylase activity, but additional factors, including stress, caffeine, smoking, and the time of day, affect salivary amylase activity (37, 42, 43). Amylase initiates the breakdown of dietary polysaccharides through the hydrolysis of internal alpha-1,4-glucosidic bonds to release smaller malto-oligosaccharides, maltotriose, maltose, and a small amount of free glucose (44, 45). Dietary starch is a form of digestible carbohydrate primarily composed of alpha-1,4-glycosidic-linked glucose moieties, and its digestion is affected by the expression of *AMY1* (46, 47). Salivary amylase is expressed in the major salivary glands (parotid, submandibular, and sublingual) in the oral cavity and increases following ingestion of food to aid in starch degradation (48, 49). Mechanical disruption of the food matrix is necessary for increasing enzymatic access, with a reduction in mastication decreasing carbohydrate digestion and absorption (46). Salivary amylase activity initiated in the oral cavity remains significant past gastric digestion, into the lower gastrointestinal (GI) tract within the food bolus (Figure 1) (50, 51).

Pancreatic amylase, encoded by *AMY2*, comprises 5–6% of the protein content in pancreatic secretions (52). It is synthesized by pancreatic acinar cells and secreted into the duodenal lumen through the pancreatic ductal system in response to a combination of vagal nervous signaling and regulatory hormonal peptide release from the small intestine (53, 54). It represents the primary amylolytic enzyme during the intestinal phase of digestion. Like salivary amylase, pancreatic amylase is an alpha-amylase, which is specific to internal alpha-1,4-glucosidic bonds. Many animals have pancreatic amylase, but no salivary amylase. Humans have both pancreatic and salivary amylase, encoded by genes located adjacent to each other on chromosome 1.

### 1.2 Structure of the amylase locus

**Figure 2:**
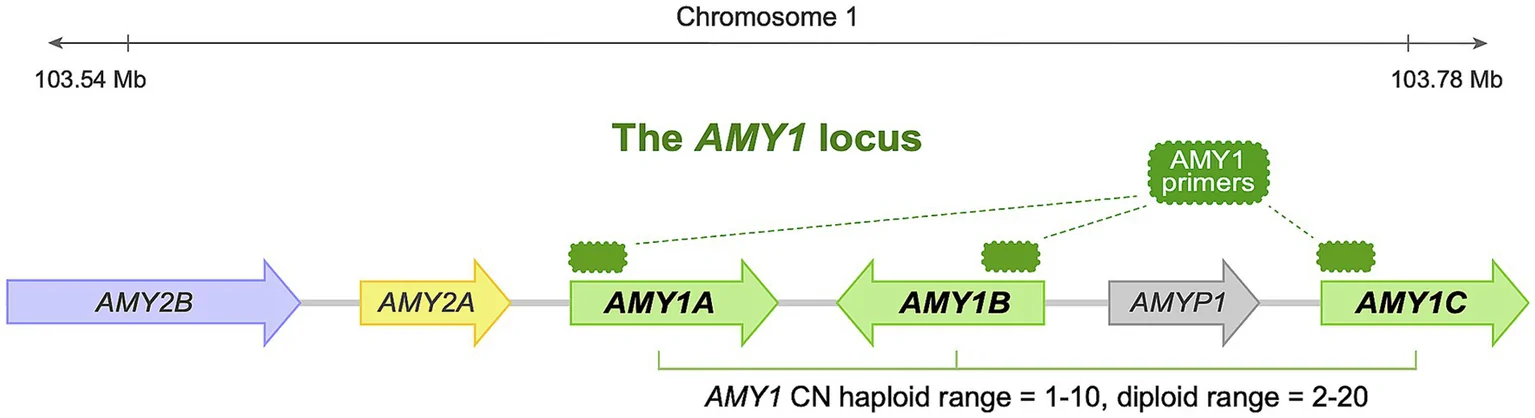
Structure of the human *AMY1* gene locus. The *AMY1* and *AMY2* genes are located adjacent to each other on human chromosome 1. *AMY1*-specific primers target *AMY1A, AMY1B,* and *AMY1C* paralogs to estimate overall *AMY1* copy number. The pancreatic amylase gene paralogs *AMY2A* and *AMY2B* are shown in yellow and purple, respectively. The nonfunctional pseudogene is depicted in gray. Arrows represent the directionality of the genes.

The human amylase locus, located on chromosome 1p21.1, contains the genes *AMY1* and *AMY2* (Figure 2). The promoter region of *AMY1* contains a gamma actin pseudogene disrupted by a retroviral-like element, which is sufficient to direct amylase expression to the parotid gland (49, 55). *AMY1* and *AMY2* are gene copy number (CN) variants. Copy number variants (CNVs) are structural variations in the genome where segments of DNA are present in variable copy numbers between individuals (56). CNVs can encompass entire genes or regulatory regions, potentially altering gene dosage and affecting gene expression. CNVs are a major source of genetic diversity and can influence phenotypic traits, disease susceptibility, and evolutionary adaptation.

The salivary amylase gene paralogs include *AMY1A*, *AMY1B*, and *AMY1C*. The salivary paralogs exhibit over 99.9% identity at the nucleotide level (57). Diploid *AMY1* CN is typically reported as the sum of the copies of *AMY1A*, *AMY1B*, and *AMY1C* in both haplotypes and ranges from 2 to 20 copies (58). *AMY2*, which encodes pancreatic amylase, is the ancestral copy of the gene from which *AMY1* originated, and the paralogs include *AMY2A* and *AMY2B*. *AMY2A* CN ranges from 0 to 6 copies, and *AMY2B* CN ranges from 2 to 7 copies. There is also a pseudogene (58), referred to as *AMYP1* or *AMY2A_p_*, an incomplete copy of *AMY2A*, which must be avoided when designing primers to quantify the intact copies of the amylase gene. The *AMY2* gene paralogs have 94% sequence identity with each other, and the *AMY1* gene paralogs are approximately 93% identical to the *AMY2* gene paralogs.

Not all humans have all *AMY1* or *AMY2* paralogs, and several groups have characterized the haplotypes of the amylase locus. The haplotypes have different combinations of the *AMY1* and *AMY2* paralogs, and some haplotypes include duplications of the paralogs. There are at least 30 documented haplotypes (59). Different populations around the world have different haplotype prevalences. Most people have an even-numbered diploid *AMY1* CN because haplotypes tend to have an odd-numbered CN of *AMY1*, and the sum of two odd numbers is an even number (57, 60). In Europeans, *AMY1* CN is correlated with *AMY2A* CN, so reported associations of *AMY1* CN with phenotype in this population could be due to salivary or pancreatic amylase. *AMY1* has greater CN variation than *AMY2* (61, 62). Nonallelic homologous recombination likely has produced *AMY1* duplications and deletions, whereas nonrecurrent microhomology-mediated break-induced replication, which occurs less frequently, is responsible for *AMY2* CN variation (59).

### 1.3 Evolutionary context of mammalian amylase

From mouse to man, amylase gene duplications appear to have occurred as an adaptation to dietary starch intake. Increases in amylase CN arose independently throughout the mammalian lineage, i.e., mice, rats, dogs, pigs, and humans, as suggested by the presence of lineage-specific retrotransposons near amylase genes (63). The ancestral amylase gene in mammals was pancreatic, and duplications of the amylase gene are found in all species with salivary expression. Thus, duplication of the gene appears to have allowed for a change in genetic architecture, facilitating expression in a different tissue, the salivary glands. Mammals consuming greater amounts of starch in their diet, typically because of domestication by humans or access to human food, have higher amylase CN accompanied by salivary amylase expression.

Haplotypes with higher *AMY1*, *AMY2A,* and *AMY2B* CN are enriched in modern-day human populations with historically agricultural diets compared to populations that historically subsisted via fishing, hunting, and pastoral activities. Estimates have timed the original duplication as having occurred 800,000 to 200,000 years ago (59, 64). Subsequently, haplotypes with amylase gene duplications were fixed during selective sweeps at different times during evolutionary history, coinciding with how easily people could access starch. For example, haplotypes with amylase duplications increased in frequency in Eurasians about 12,000 years ago with the development of agriculture in the Crescent Valley. Expansion in indigenous Peruvian Andeans occurred between 6,000 and 10,000 years ago, coinciding with the domestication of the potato, and resulting in enrichment of the highest *AMY1* CNs in the world (65). By contrast, in some populations with low-starch diets, haplotypes with deletions became more prevalent. *AMY2A* is frequently missing in Siberians, who historically hunted mammoth and reindeer with limited access to plant carbohydrates (62).

These findings support a model of genetic convergence followed by positive selection in humans, whereby diet-driven selective pressures have repeatedly shaped the amylase locus, illustrating how gene CN variation can facilitate rapid adaptation to changing ecological niches and diets. A remaining question is how increased *AMY1* CN impacts metabolic health in modern times. The main objective of this review is to assess the impact of *AMY1* CN on adiposity and glucose metabolism because many studies have addressed this question, and the results have been conflicting. Because the gut microbiota produces metabolites that contribute to metabolic health, we will also consider studies reporting associations between *AMY1* CN and gut microbiome composition.

## 2 Methods

We searched PubMed, Web of Science, Cumulative Index to Nursing and Allied Health Literature, and Centre for Agricultural and Biosciences International for articles focused on salivary amylase gene copy number and one or more of the following: BMI, glucose metabolism, and the microbiome. (See search terms used for each database in Supplementary Table S1.) The search was initially conducted on 08/10/2022 and updated on 09/13/2024.

**Figure 3:**
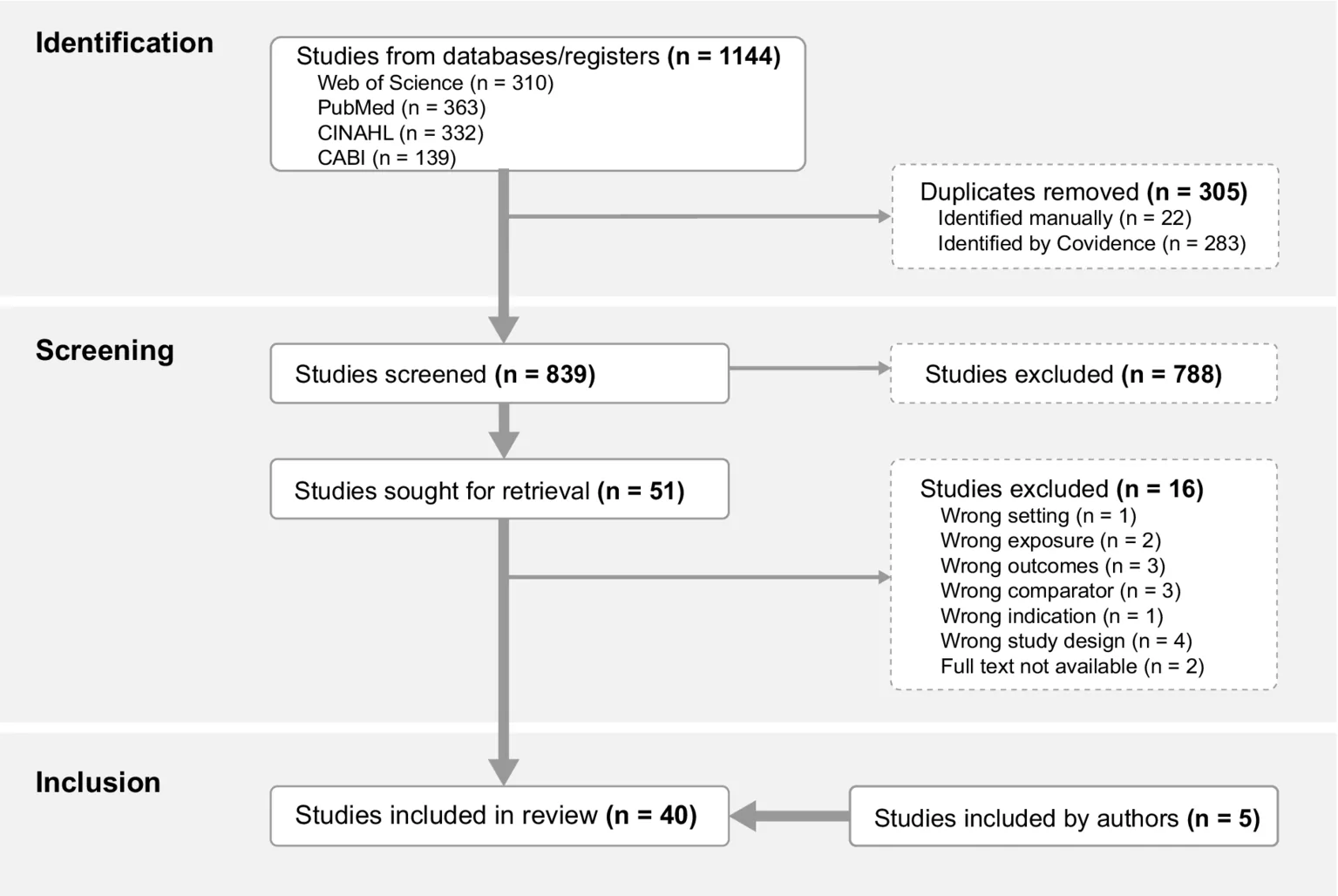
PRISMA flow diagram. This diagram summarizes the workflow for article identification, screening, and inclusion for this review.

After duplicates were removed, the results from all searches were screened using eligibility criteria. Two researchers independently conducted a title and abstract review, and then a full-text screening. The studies were included in this manuscript if they were primary research articles, written in English, had been peer-reviewed, and if the content of the text was relevant to the topic “Impact of *AMY1* gene copy number variation on adiposity, glucose homeostasis, and metabolic health.” Articles were excluded if the study population was children or adolescents aged below 18 years; the study was conducted in animal models; the study only examined the impact of salivary amylase quantity and activity in saliva but did not estimate *AMY1* copy number; the study only examined SNPs in the *AMY1* gene, but not the copy number. Review papers, abstracts, posters, and articles without a published full text were also excluded. The abstract screening yielded 51 results. Thirty-five articles remained after the full-text screening, which were included in this review. Five more relevant articles previously known by the authors were added to the list of articles for a total of 40. All included articles were published between 2012 and 2024. Following inclusion, the relevant study characteristics were extracted and utilized in the interpretive analysis. The full screening workflow can be seen in Figure 3.

## 3 Discussion

### 3.1 Association between *AMY1* CN and adiposity

Factors affecting food accessibility, such as famine and agricultural innovations, have provided selective pressure throughout history. *AMY1* CN varies with the historic diet in mammals, including humans. An enhanced capacity to digest starch as a source of energy would provide a survival advantage when food is scarce. However, in an environment with unprecedented access to foods containing digestible carbohydrates, excess calories could result in weight gain, leading to metabolic syndrome. For these reasons, researchers hypothesized that increased *AMY1* CN could predispose one to excess body weight in modern times.

Researchers have tested this hypothesis using several metrics of adiposity: body mass index (BMI), body fat percentage, and categorization–people with or without obesity (Table 1; Supplementary Table S2). Falchi et al. (66) were one of the first groups to assess the association between *AMY1* CN and adiposity, and reported that *AMY1* CN is inversely correlated with BMI. This finding is counterintuitive because *AMY1* CN is positively correlated with salivary amylase activity, and more efficient starch breakdown would presumably lead to higher caloric absorption and increase body weight. Soon after, Usher et al. reported that there was no association (60). It was posited that the genotyping method used by Falchi et al. was inaccurate, with one piece of evidence being that most of the reported diploid *AMY1* CN values were odd-numbered in contrast to most people, including Europeans, who have an even-numbered diploid *AMY1* CN (57, 67).

Methods used to estimate gene CN include sequence read depth, quantitative PCR (qPCR), droplet digital PCR (ddPCR), fluorescence *in situ* hybridization, and paralog ratio testing. Gene CN measurement can be prone to error, and the relative accuracy of different genotyping methods has been debated (68–71). Bonnefond et al. (72) used ddPCR to recalculate the *AMY1* CN of the European DESIR cohort studied in the Falchi et al. manuscript and reported a nominal inverse association between *AMY1* CN and BMI in adults. In this analysis using ddPCR, they also observed more even diploid *AMY1* CNs in the dataset than odd, as expected based on other studies.

At least 20 additional studies have addressed this question in adult populations globally. Eight studies reported no association between *AMY1* CN and adiposity. Ten studies reported an inverse association, and in six of these, the association was conditional, e.g., only detected in individuals who are females with obesity, of Arab ethnicity, with high starch intake, or with *AMY1* CN greater than 10. One of the studies reporting an inverse association had only female participants and a confounding factor, i.e., energy intake was higher in those with lower *AMY1* CN. Two studies reported a positive association between *AMY1* CN and adiposity, with one only detecting a positive association in individuals with high starch intake.

Collectively, these reports suggest that if there is an association between *AMY1* CN and adiposity, there are other factors influencing individual outcomes. Many of these studies utilized large datasets comprising hundreds or thousands of individuals. However, only three of these studies addressed habitual dietary intake as part of their analysis. It stands to reason that a participant’s habitual intake of starch, the substrate of the amylase enzyme, could be a factor.

Additionally, people with different ancestries could have other genetic polymorphisms that enhance or counteract the effects of *AMY1* CN. There could even be a threshold of a required CN before physiological effects are clinically meaningful, potentially confounding the conclusion of the eight studies that categorized high and low *AMY1* CN based on population medians. At present, without knowing more about the relative influence of *AMY1* CN and other etiological factors contributing to obesity, as well as the underlying mechanisms, it is not reliable to use *AMY1* CN as a sole predictor of obesity risk.

### 3.2 Association between *AMY1* CN and postprandial glucose response to starch

Several groups have tested the association between *AMY1* CN and blood glucose changes in response to carbohydrate consumption (Table 2; Supplementary Table S3). Postprandial glucose response (PPGR) is the change in blood glucose following food and drink intake. PPGR is influenced by meal composition and timing, lifestyle factors including physical activity, and individual traits (i.e., baseline metabolic state, insulin sensitivity, genetics, and gut microbiome composition). Amylase inhibitors have been studied as a potential therapeutic approach to decrease PPGR (73, 74). Since salivary amylase targets digestible starch, and *AMY1* CN is positively correlated with salivary amylase activity (SAA), one would expect a positive association between *AMY1* CN and the increase in blood glucose following starch consumption.

Salivary amylase activity is a major determinant of the rate at which digestible polysaccharides are hydrolyzed and absorbed. Up to 30% of cooked starches are liberated to disaccharides or small oligosaccharides within 30 s of ingestion, and up to 80% of simple starch can be degraded by the time the bolus reaches the small intestine (45, 47) (Figure 1). Following gastric emptying, pancreatic amylolytic exocrine secretions, including both salivary and pancreatic amylase isozymes, progress starch digestion to maltose, maltotriose, and *α*-dextrins (75, 76). These small glucose polymers are completely hydrolyzed by brush border-associated enzymes to liberate free glucose, which is subsequently taken up by the sodium-glucose cotransporter 1 (SGLT1) and glucose transporter 2 (GLUT2) transporters into the enterocyte (77). This transport system is highly adaptive to the amount of available carbohydrates ingested, so the absorption rate is limited mostly by the availability of oligosaccharides and disaccharides reaching the brush border (77). Therefore, high *AMY1* CN would intuitively increase the rate and absolute amount of glucose absorbed proximally in the digestive tract, and subsequently the postprandial rise in blood glucose after starch consumption.

At least four studies have reported higher postprandial glucose responses to starches by individuals with high *AMY1* CN compared to those with low CN. Atkinson et al. (78) found *AMY1* CN accounted for 26–61% of the relative glycemic response to six starchy foods (25–50 g available carbohydrates) compared to glucose, with no differences observed between high and low *AMY1* CN groups after ingestion of sucrose or fructose. Barber et al. (79) also observed a greater increase in postprandial blood glucose in people with high *AMY1* CN despite lower fasting blood glucose, an observation repeated by Zhang et al. (80) Farrell et al. (81) found a significant difference in blood glucose between high and low CN groups after consuming 40 g of starch, although there was no significant difference if 80 g of starch was consumed.

Several groups observed a negative association between *AMY1* CN and PPGR, or no significant effect. Mandel and Breslin (82) found a significantly attenuated PPGR in individuals with a high *AMY1* CN compared to those with a low CN following corn starch but not glucose ingestion. Tan et al. (83) and Alberti et al. (84) found no significant association between *AMY1* CN and PPGR to starch consumption. No overall difference in blood glucose between high and low *AMY1* CN groups was observed by Higuchi et al. (85) when testing healthy Japanese women; however, blood glucose was significantly higher at 60 min in the low *AMY1* CN group compared with the high group, suggesting a delayed response.

Researchers who observed a higher PPGR response to starch by low *AMY1* CN individuals compared with high CN individuals proposed physiological mechanisms. Mandel and Breslin (82) attributed their results to an absence of cephalic phase, or preabsorptive, insulin release in people with a low *AMY1* CN as observed in their study population. Cephalic phase insulin release (CPIR) is a preabsorptive response to food mediated mostly by autonomic nervous signaling before and during food ingestion, lasting approximately 10 min (86). This early secretion only accounts for 1–3% of total insulin released following a meal but has a significant influence in preparing the body to digest, absorb, and store carbohydrates efficiently (87). Thus, a greater CPIR attenuates postprandial increases in blood glucose (87). The more active glycosylated isoform of salivary amylase has been demonstrated to completely hydrolyze malto-oligosaccharides to glucose (88). The presence of glucose in the oral cavity would theoretically be higher following starch intake in individuals with high *AMY1* CN, stimulating the SGLT1 and GLUT2 glucose transporters in taste receptor cells (89). This would activate CPIR through nervous system-mediated signaling (82). However, Farrell et al. observed no difference between high and low *AMY1* CN groups in the CPIR response, but they had notable differences in study design, including the use of white wheat bread as opposed to corn starch in solution (81).

Barling et al. (90) proposed another mechanism to explain a higher PPGR to starch by individuals with low salivary amylase activity. They postulated that increased carbohydrate digestion would lead to a greater release of the incretin hormone, gastric inhibitory polypeptide (GIP), in the proximal duodenum immediately following gastric emptying. Incretin hormones are potent stimulators of pancreatic insulin release, accounting for the majority of the post-prandial insulin secretion (91). Incretin hormone secretion is promoted by the stimulation of SGLT1 with glucose or maltose (92). High salivary amylase activity could increase the stimulation of these transporters through earlier glucose absorption in the proximal duodenum, leading to a prompt insulin-driven clearance of glucose from circulation through GIP signaling. By contrast, there would be a delayed incretin response in low *AMY1* individuals reliant on another incretin hormone, glucagon-like peptide-1 (GLP-1), released from more distally located L-type cells, resulting in a prolonged blood glucose response. Although incretin hormones could explain the greater PPGR in individuals with low salivary amylase activity following starch ingestion observed in their study, neither GIP nor GLP-1 was measured.

When addressing this hypothesis, the study by Atkinson et al. (78) included a relatively large sample size of 114 individuals, both sexes, and European Caucasian and Asian adults. Although some of the *AMY1* CN measurements were made using quantitative PCR, steps were taken to confirm the validity of their assay by comparing with droplet digital PCR measurements. Their study design tested six starch-rich foods: white bread, boiled potato, basmati rice, rolled oat porridge, pasta, and cornflakes cereal. In their statistical model, *AMY1* CN was used as a continuous variable as opposed to the categories high and low *AMY1* CN, which have variable thresholds between studies. These aspects address several of the limitations of studies finding inverse or no association. At least three smaller studies are in general agreement with their conclusions (79–81). Evidence most strongly supports that high *AMY1* CN individuals have a higher PPGR to digestible starch than low *AMY1* CN individuals.

### 3.3 Association between *AMY1* CN and long-term glucose homeostasis

Research groups have tested the association between *AMY1* CN and indicators of long-term glycemic control: insulin resistance, insulin sensitivity, hemoglobin A1c (HbA1c), and type 2 diabetes status (Table 2; Supplementary Table S3). If a high *AMY1* CN is associated with a chronically higher PPGR to starchy foods than a low *AMY1* CN, high *AMY1* CN individuals may be more likely to develop insulin resistance and, consequently, type 2 diabetes (T2D). If so, a high *AMY1* CN could be considered a risk factor for aberrant glucose homeostasis.

Counterintuitively, multiple studies have reported a protective effect of a high *AMY1* CN. Liu et al. (93) observed that a low *AMY1* CN was associated with increased insulin resistance and risk of T2D with age. Higuchi et al. (85) reported that low *AMY1* CN was associated with HbA1c ≥ 5.4% in a cohort of healthy Japanese women. Zhang et al. (80) found a significantly elevated fasting insulin and homeostasis model assessment of insulin resistance (HOMA-IR) associated with a low *AMY1* CN. Choi et al. (94) reported similar findings—an inverse association between HOMA-IR and *AMY1* CN in Korean men. Barber et al. (79) observed a positive correlation between *AMY1* CN and the oral glucose sensitivity score but no significant association with homeostasis model assessment of insulin resistance (HOMA-IR) in a study of 17 individuals. The sample sizes for the largest studies, Liu et al. (93) and Choi et al. (94), were 1,729 and 1,257, respectively. Choi et al. studied Korean men, and Liu et al. used data from Puerto Ricans and Europeans.

Other studies reported interaction effects or associations suggesting that a high *AMY1* CN increases the risk of aberrant glucose homeostasis or has no effect. Hamid et al. (95) reported an interaction between *AMY1* CN and starch intake on HOMA-IR in a large Swedish cohort. The negative association between starch intake and HOMA-IR (and T2D) was greater in individuals with *AMY1* CN ≥ 10. This finding was corroborated by the same team in another study population (81). When examining individuals with a high *AMY1* CN, the lowest fasting blood glucose coincided with a high starch intake, and the highest fasting glucose with a low starch intake. Valsesia et al. (96) observed no interaction between diet, *AMY1* CN, and glucose homeostasis at baseline or following weight loss in a large retrospective study (96). This study population consisted of individuals with overweight or obesity, so adiposity may have been a confounding factor. Atkinson et al. (78), despite observing higher PPGR to starchy foods by individuals with high *AMY1* CN, found no overall association with fasting plasma glucose or HOMA-IR in their prospective cohort study. Lv et al. (97) also found no association with T2D in a Chinese cohort of 60. Although a study conducted on a Qatari cohort showed no association between *AMY1* CN and glucose homeostasis, high salivary amylase activity had a significant protective effect against diabetes (98). Hasegawa et al. (99) reported that *AMY1* CN is positively associated with diabetes and HbA1c. The mean *AMY1* CN in this study population was 10.3, which is higher than the mean values reported in other populations, e.g., Qatar, Japan, the United States, and Mexico. They posited that high *AMY1* CN in the Northern Japanese population may have provided an evolutionary benefit resulting in positive selection, but their exceptionally high daily starch intake during recent times has an overwhelming effect.

Most studies detect an association between *AMY1* CN and long-term glucose homeostasis, but the direction of the effect is conflicting. Based on the effect size of *AMY1* CN on HOMA-IR derived from the parameter estimates of the statistical model used by Choi et al. (94), you would need several thousand study participants to have 80% power to detect a significant effect with *p* < 0.05, so smaller studies may have lacked sufficient power. If there is a positive association between *AMY1* CN and aberrant glucose homeostasis, the explanation is straightforward—individuals with high *AMY1* CN are at higher risk for T2D due to chronic exposure to higher circulating glucose. If high *AMY* CN is protective against insulin resistance and T2D, the mechanism would be more complicated.

**Figure 4:**
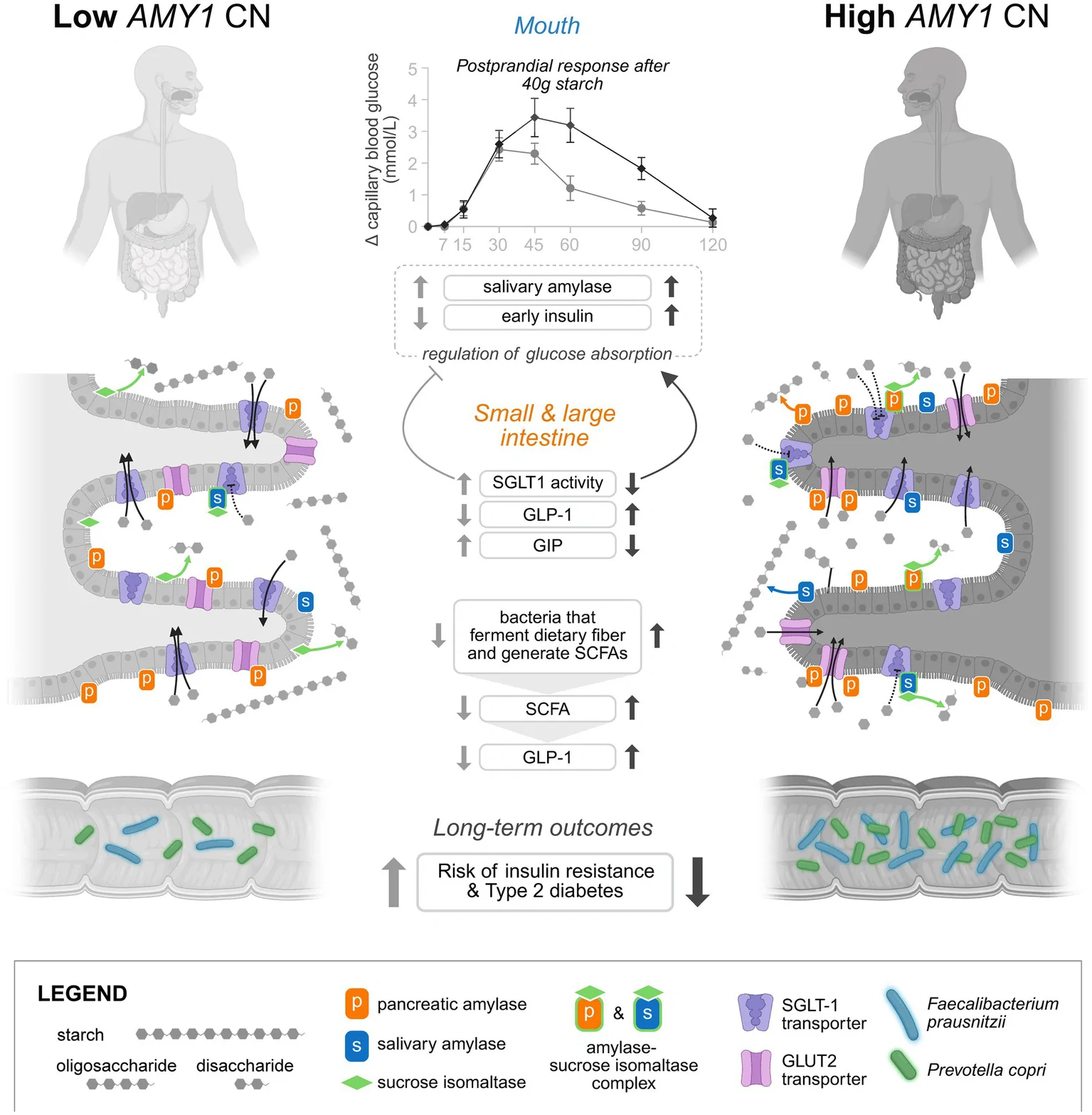
Proposed model for the metabolic benefit of a high *AMY1* copy number. Lighter gray is associated with low *AMY1* CN in the figure, and darker gray is associated with high *AMY1* CN. In the proposed model, high salivary amylase contributes to increased early breakdown of digestible starch, resulting in an initial rise in blood glucose. This would cause increased early insulin release compared to *AMY1* low individuals, and a subsequent greater increase in salivary amylase. Amylase binds to sucrase-isomaltase and SGLT1 transporters, reducing the activity of SGLT1 and shifting glucose absorption toward the GLUT2 transporters that translocate from the basolateral surface to the apical surface during high carbohydrate conditions. This increases the ratio of GLP-1 to GIP, contributing to greater regulation of glucose absorption and appetite in high *AMY1* CN individuals compared to low *AMY1* individuals. Early insulin secretion also regulates the amount and duration of GLUT2 transporters at the apical membrane, further contributing to the regulation of glucose absorption. This could prevent excessive rapid internalization of glucose following a high starch dose. High salivary amylase also changes the composition of carbohydrates (a higher proportion of dietary fiber than simple carbohydrates) that reach the large intestine, contributing to an enrichment of beneficial bacteria that promote SCFA production, such *as P. copri* and *F. prausnitzii*. SCFAs are beneficial metabolites that can also further increase GLP-1 secretion when absorbed. Over time, these mechanisms mediated by salivary amylase could decrease the risk of metabolic disorders like insulin resistance and type 2 diabetes. Abbreviations: SGLT1: sodium glucose cotransporter 1; GLUT2: glucose transporter 2; SCFA: short-chain fatty acids; GIP: gastric inhibitory polypeptide; GLP-1: glucagon-like peptide-1; CN: copy number. Created in BioRender. Poole, A. (https://BioRender.com/16im9jj) is licensed under CC BY 4.0.

One potential mechanism involves a regulatory role of salivary amylase in glucose absorption via SGLT1 (Figure 4). This implicates the involvement of an additional enzyme because amylase breaks down starch to oligosaccharides, but SGLT1 will only absorb monosaccharides. Amylase binds to *N*-linked glycans of glycoproteins at the duodenal enterocyte brush border (100). The main substrates of the amylase enzyme were determined to be SGLT1 as well as sucrase-isomaltase, an enzyme chiefly responsible for the breakdown of maltose to liberate free glucose. Upon binding, the enzymatic activity of amylase and sucrase-isomaltase increases by 240 and 175%, respectively (100). However, at the same time, high amylase concentrations drastically reduced the rate of glucose uptake by SGLT1. *N*-glycoside binding also promoted amylase uptake and degradation within enterocyte lysosomes (101). Mechanistically, amylase could serve to protect enterocytes from rapid glucose influx while continuing to degrade starch. The slower internalization and degradation of salivary amylase could allow the gradual return of normative SGLT1 activity and glucose uptake while dampening postprandial blood glucose spikes following a large starch bolus.

GIP release in the small intestine may also play a role (Figure 4). GIP is a key contributor to postprandial insulin secretion (44%) (102). As postulated by Barling et al., increased early stimulation of SGLT1 receptors in the proximal duodenum in individuals with a high *AMY1* CN could accelerate increases in GIP and insulin, allowing for more efficient glucose utilization (90). The release of GIP and GLP-1 is directly related to the glucose absorption rate, so delayed glucose uptake would delay the incretin effect (103). GIP can directly stimulate enterocyte uptake of glucose through the accumulation of cyclic adenosine monophosphate mediated by SGLT1 receptors. Increased salivary amylase activity may counter GIP’s effect (raising blood glucose concentrations) by regulating the SGLT1-mediated glucose uptake in the intestine. In GIP receptor knockout mice, beta cell sensitivity was increased during a glucose challenge, and fasting glucose was decreased, while GLP-1 promoted beta cell survival and inhibited apoptosis (104). GIP receptor inhibition leads to resistance to weight gain and glucose dysregulation in rodent models of aging and obesity (105, 106). GIP is mediated by SGLT1 transporters but not GLUT2 transporters in enteroendocrine cells (107). GLP-1 secretion is more reliant on the GLUT2 transporter, which needs a greater concentration of glucose in the lumen for its activation. Inhibition of SGLT1 may selectively decrease GIP secretion and SGLT1 glucose absorption while promoting greater GLP-1 release. Salivary amylase activity may increase to prevent further absorption and/or inhibit gluconeogenic pathways stimulated by GIP. If starch degradation is slower in low *AMY1* individuals and SGLT1 is unchanged, this could extend the window of incretin hormone secretion following a meal. Over time, prolonged release of GIP could contribute to pancreatic beta cell fatigue and a reduced postprandial incretin response. To our knowledge, there have been no studies comparing incretin responses in people with high and low *AMY1* CN on a diet high in starch.

Pierzynowski et al. (108), through a series of experiments, described an intra-pancreatic axis of communication in which endocrine secretions directly influence exocrine secretions and vice versa, termed the halo phenomenon. Oral and intravenous administration of amylase significantly lowered the postprandial insulin response in a healthy porcine model, while the blood glucose response was attenuated in a model of diabetes independent of insulin (109, 110). They stipulate that both insulin and amylase are induced through food, and insulin subsequently increases amylase through the halo phenomenon. Amylase then regulates both glucose absorption and insulin to prevent an excessive hyperglycemic and hyperinsulinemic response, thus preventing pancreatic islet fatigue and glucose insensitivity (111). While these trials used pancreatic amylase, it would be reasonable to hypothesize that salivary amylase would possess similar signaling functionality. Following insulin infusion in rats, acute phosphorylation of the insulin receptor in the parotid gland (the main site of postprandial amylase expression) was similar to that of the liver at 30 s (112). Because the liver is a primary postprandial target for insulin, this suggests postprandial salivary amylase release may be directly linked to insulin signaling in a potential feedback mechanism. Observations in patients with type 2 diabetes provide additional insight. Fasting salivary amylase activity is generally found to be higher in people with diabetes (113), even though there is reduced saliva production and secretion (114). We observed that individuals with T2D or prediabetes had a greater increase in salivary amylase activity (43% versus 14%) per each additional copy of *AMY1* than individuals who self-reported being without T2D or prediabetes (70). A higher salivary amylase activity would stimulate this hypothetical regulatory pathway and counteract the dysregulation occurring in T2D.

In summary, the effects of *AMY1* CN on long-term glucose homeostasis are incompletely understood. Underlying mechanisms may involve the effects of salivary amylase activity on glucose transporters and/or incretin hormones. The disagreement in findings between studies could be due to biological, dietary, and/or environmental factors in addition to technical aspects, i.e., genotyping methods and choice of glucose metabolism assays. The populations studied encompass differing BMI ranges, habitual starch intake, sex ratios, ancestry, and high and low *AMY1* group designations. To help address reproducibility, future studies could include these variables as covariates in the statistical models used to test for associations between *AMY1* CN and metabolic health parameters. This would be difficult to achieve with smaller, individual studies, so one approach could be to obtain measures (e.g., fasting blood glucose, HOMA-IR, T2D status) that do not require an intervention and combine the data in a meta-analysis. Two additional (more complex) factors that could modify the associations include other genes that contribute to metabolic health and gut microbiome composition.

### 3.4 Association between *AMY1* CN and the gut microbiome

The human gut is colonized by trillions of microbes collectively termed the microbiota (115). The microbiota possesses more genes than the human host, and the summation of these genes is referred to as the microbiome. Gut microbiota composition is shaped by intrinsic and extrinsic factors, including mode of birth, geographical location, lifestyle, genetics, medications, and diet. The gut microbiota has a profound impact on human health, including roles in obesity, glycemic control, and other physiological processes, through a variety of mechanisms (116). The microbiota exerts effects within the human body either through direct membrane signaling or postbiotic metabolites, including short-chain fatty acids (SCFAs). Often, the result is alterations in gene expression that either directly or indirectly alter host physiology. Alternatively, these metabolites can be directly absorbed by the host and utilized for energy (117). Due to the malleability of the gut microbiota and its potential to produce metabolites that affect host physiology, targeting the gut microbiota through diet or pharmaceuticals to benefit metabolic health is a strategy that is currently being pursued (118).

Undigested carbohydrates are used as a major selective driver of microbiota community composition, which determines community function and the resulting impact on metabolic health (119). Salivary amylase activity could influence the carbohydrate composition of a food bolus before it reaches the human colon, with theoretically more digestible starch reaching the colon in individuals with a low *AMY1* CN than in those with a high *AMY1* CN. Differences in the complex carbohydrate substrates presented to the gut microbiota could differentially affect the metabolites produced that influence adiposity and glycemic control.

Gut microbes break down complex carbohydrates via specialized machinery, including carbohydrate-active enzymes (CAZymes) and polysaccharide utilization loci (PULs), which are gene clusters encoding enzymes, transporters, and binding proteins for efficient carbohydrate metabolism (120). There are five classes of CAZymes: glycoside hydrolases, glucosyl transferases, polysaccharide lyases, carbohydrate esterases, and auxiliary activities. The CAZymes present determine a microbe’s capability to degrade a particular complex carbohydrate substrate. Examples of bacteria containing CAZyme-encoding genes include *Bacteroides*, *Prevotella* spp. (commonly enriched in individuals consuming plant-based diets), and Ruminococcaceae, e.g., *Ruminococcus bromii*, a keystone degrader of resistant starch in the human gut (121). These complex carbohydrate degraders contribute to the production of host-beneficial metabolites like SCFAs and supply downstream substrates that support the wider microbial community.

At least six groups have identified microbial taxa and genes associated with *AMY1* CN (Table 3; Supplementary Table S4). Using shotgun metagenomic sequencing on fecal DNA, Poole et al. observed significant enrichment of two CAZyme classes—glycoside hydrolases and polysaccharide lyases—in low *AMY1* CN (CN < 5) individuals compared to high *AMY1 CN* individuals (CN > 8) (122). There was a similar trend for two additional CAZymes classes, carbohydrate esterases and auxiliary activities. This suggests that more complex carbohydrates escape digestion in the small intestine and reach the colon to support the gut microbiota in low *AMY1* CN individuals than in high CN individuals. There were eight species of *Bacteroides* identified in fecal samples in the study, and seven of these had significantly higher read counts in high *AMY1* CN individuals, whereas a single species of *Bacteroides*, *B. cellulosilyticus*, had higher read counts in low *AMY1* CN individuals. In addition, gene families classified as *Ruminococcus*, including *Ruminococcus bromii*, had higher read counts in high *AMY1* CN individuals, as well as *Prevotella copri*. In agreement, Leon-Mamila et al. (123) observed that *AMY1* CN was positively correlated with the relative abundance of *Prevotella* in adults in Mexico; *P. copri* was enriched two-fold among those with *AMY1* CN ≥ 10 when compared to individuals with CN ≤ 4. These findings imply that gut microbiota in high versus low *AMY1* CN individuals are specialized to degrade certain forms of carbohydrates, possibly due to modification by salivary and/or pancreatic amylase. *AMY1* CN was correlated with *AMY2* CN in the study participants (122), so pancreatic amylase could be involved.

In another study employing shotgun metagenomic sequencing, Hasegawa et al. (99) observed that *AMY1* CN was negatively associated with the abundance of the KEGG (Kyoto Encyclopedia of Genes and Genomes) pathway for acarbose and validamycin biosynthesis. Acarbose is an antidiabetic drug that decreases PPGR through alpha-glucosidase inhibition (124). They posited that this mechanism results in higher glycemia in those with a high *AMY1* CN, resulting in increased HbA1c and weight gain. They also noted an enrichment of the genus *Dialister* with increasing *AMY1* CN in males, while a negative association between *AMY1* CN and the relative abundance of *Dialister* was noted in females. *Dialister* is known to consume succinate as a growth substrate, interacts with *Bacteroides thetaiotaomicron* to produce propionate, and has been linked to inflammation, metabolic health, and several diseases (125). Enrichment of *Dialister* has been associated with the failure to lose weight, lower insulin sensitivity, and increased visceral fat (126–128). *Dialister hominis*, a species found in the human gut, contains CAZymes specified in the CAZy database, suggesting the potential to degrade complex carbohydrates (129). At least two research teams reported no differences in microbiome composition depending on *AMY1* CN. One team used fluorescence *in situ* hybridization and bacterial enumeration by flow cytometry (79), and the other sequenced the hypervariable region V1 to V3 of the 16S rRNA gene (81). These methods may have limited the ability to detect species associated with *AMY1* CN.

Researchers also addressed whether the prevalence of the kingdom Archaea in the gut microbiota is associated with *AMY1* CN. Atkinson et al. (78) observed that low *AMY1* CN (CN ≤ 4) individuals had significantly higher breath methane at baseline compared to high *AMY1* CN (CN ≥ 10) individuals. The total methane excreted over 8 hours following consumption of white rice with or without added resistant starch was also higher in low *AMY1* CN (CN ≤ 4) individuals. In agreement, another study observed a trend for elevated breath methane in individuals with low salivary amylase activity compared to individuals with high salivary amylase activity following rice consumption (130). Atkinson et al. postulated that low *AMY1* CN favored the enrichment of methane-producing Archaea, such as *Methanobrevibacter smithii*. Methanogens in the gut have been linked to BMI, inflammatory bowel disease, and other conditions; so, if an association with *AMY1* CN is confirmed, there could be health-related implications. Neither of these studies sequenced the gut microbiome, and studies with shotgun metagenomics sequencing data from microbial communities did not report that the relative abundance of members from Archaea was associated with *AMY1* CN. Discordance between the findings could be because Archaea vary in prevalence and abundance depending on the population demographics and detection methods used (131, 132).

In Poole et al., there were experiments performed in addition to shotgun metagenomics data analysis to assess functional differences between the gut microbiota of high and low *AMY1* CN groups (122). Germ-free mice inoculated with stool from human donors with high *AMY1* CN had higher adiposity than mice receiving stool from donors with low *AMY1* CN. This suggests increased accessibility to polysaccharide-derived calories facilitated by a microbiome shaped by high *AMY1* CN. Unlike humans, mice have a fixed *AMY1* CN, so this result may not translate to microbial-influenced weight gain in humans, who would have a concomitant higher salivary amylase activity. But it substantiates the potential of *AMY1* CN to modulate the functional capacity of the gut microbiota. Poole et al. also reported that fecal SCFA concentrations were predictive of whether the participant had high or low salivary amylase activity (SAA). SAA group (high versus low) was determined using k-means clustering on mean salivary amylase activity measurements. Fecal concentrations of acetate, butyrate, and propionate were higher in participants with high SAA than in those with low SAA. Since SCFAs in the colon can stimulate secretion of GLP-1 to lower blood glucose concentrations (133, 134), this finding supports the possibility that the gut microbiota in people with high *AMY1* CN promotes insulin sensitivity (Figure 4).

Hjorth et al. (135) found that a high *Prevotella*-to-*Bacteroides* (P/B) ratio predicted significantly greater weight loss when participants were following the New Nordic Diet, a dietary strategy focusing on the consumption of dietary fiber, whole grains, fruits, and vegetables. However, this prediction only applied to those with low *AMY1* CNs, whereas no association was found in individuals with *AMY1* CN > 6.5 copies. The P/B ratio at baseline was not associated with *AMY1* CN, although all participants with undetectable *Prevotella* (0-*Prevotella*) had high *AMY1* CN. In an earlier study by this group using the same sample cohort, the group of 0-*Prevotella* was found to lose as much weight as the high P/B ratio group (136).

Data from two independent studies with whole grain interventions are in general agreement with their findings (137). *Prevotella* abundance at baseline was positively correlated with the decrease in fat percentage during the dietary intervention, but only in those with low *AMY1* CN. The association disappeared when participants with undetectable *Prevotella* (0-*Prevotella*) were included in the secondary analysis. Similarly, an earlier paper by this research team showed the 0-*Prevotella* group lost significantly more weight on the whole grain diet, independent of *AMY1* CN (138). Another research group observed that *AMY1* CN alone was not a predictor of weight loss (96). Using a combination of *AMY1* CN and P/B ratio to predict weight loss resulting from a whole grain diet appears accurate for individuals with low *AMY1* CN. The finding that a higher P/B ratio results in more weight loss, and 0-*Prevotella* also results in weight loss seems incongruent. There may be other bacteria contributing to the outcome in people with high *AMY1* CN and/or 0-*Prevotella*.

Our group tested *AMY1* CN as a predictor of gut microbiome changes in response to dietary supplementation with resistant starch (RS) (139). The crossover study consisted of ten-day treatment periods with each of three starches: resistant starch type 2 (RS2; HI-MAIZE® 260 starch, Ingredion), resistant starch type 4 (RS4; VERSAFIBE™ 1,490 starch, Ingredion), and corn starch (AMIOCA™ TF starch, Ingredion), which is a digestible starch–digestible by host enzymes, including amylase. We hypothesized that an *AMY1* metric (*AMY1* CN, *AMY1* group, or salivary amylase activity) would predict changes in gut microbiota composition and function in response to RS because several taxa that degrade RS had higher relative abundances in individuals with high *AMY1* CN than in those with low *AMY1* CN (122). We observed that salivary amylase activity was predictive of the abundance of *Sutterella* at the end of the digestible starch treatment. Several species of *Sutterella* have CAZymes identified in the CAZy database, so the *Sutterella* that we identified may metabolize breakdown products of host amylase. None of the *AMY1* metrics was predictive of changes in either microbiome composition or fecal SCFA concentrations in response to the RS treatments. This study was limited by the taxonomic resolution possible with 16S rRNA gene survey, and fecal SCFA concentrations may not accurately reflect the amount of SCFAs produced by the colonic microbiota (139).

In summary, researchers have observed differences in the taxa and functions of gut microbiota associated with *AMY1* CN or *AMY1* group. Only two studies used shotgun metagenomics, which provides greater taxonomic resolution and functional gene annotation. Continuing advances in sequencing technology and computational tools may facilitate more frequent use of shotgun metagenomics to provide greater insight into the relationship between *AMY1* CN and the gut microbiome in future work. The gut microbiota may partly mediate the effects of *AMY1* CN on metabolic health. Microbial metabolites, such as SCFAs, trigger signaling pathways that affect insulin sensitivity and satiety. Some studies have shown that *AMY1* CN in combination with bacterial abundances predicts weight loss outcomes in a subset of individuals, but there have been few studies that test the ability of *AMY1* metrics to predict metabolic health or microbiome-related outcomes in response to dietary interventions. Additional studies are required to further characterize the impact of amylase gene dosage on the gut microbiota and metabolic health before it is feasible to develop clinical applications.

## 4 Limitations of the current literature

Several limitations of the current body of literature potentially contribute to the heterogeneous results. As previously mentioned, CN estimation techniques can be error-prone, and there is disagreement about the accuracy and reproducibility of certain methods. Other related issues are heterogeneity in study population, biological sample type, and DNA extraction method. A CN variation of one to two copies can become problematic when the designation of high *AMY1* CN or low *AMY1* CN is based on population median values, which is the case in half (20/40) of the included studies. Physiological variation due to *AMY1* CN could be missed if CN is not analyzed as a continuous variable. There could be a threshold at which *AMY1* CN becomes clinically relevant, and this threshold may not be the median value across populations. It is possible that the relationship between *AMY1* CN and metabolic health parameters is nonlinear. Additional confounding factors include genetic background and dietary habits.

Many of the included studies do not measure SAA, which may be relevant because SAA changes in response to environmental and intrinsic factors, including time of day, stress, caffeine, smoking, and health status. SAA may be altered in obesity and T2D, with some groups reporting a sex specific effect. Future controlled studies focusing on associations between SAA and incretin responses in cases of metabolic disorder would provide valuable insight into the underlying mechanisms, as the previous feeding trials involved healthy participants. Habitual dietary intake is another important variable that is rarely included in analyses. The effect of SAA could be different for a population consuming low or moderate amounts of carbohydrates compared to those who consume high-carbohydrate diets, consistent with the evolutionary history of *AMY1*.

## 5 Future directions

Not enough data exists to use *AMY1* CN as a biomarker. Doing so may require an algorithm that takes into account genetic ancestry and historical diet. Databases coupled with biobanks like those associated with the NIH All of Us Research Program and the TwinsUK could be useful for large-scale testing of associations between *AMY1* CN and metabolic health parameters. Researchers could use blood or saliva samples to accurately determine *AMY1* CN and test for associations with cardiometabolic markers documented in electronic health records. The inclusion of various racial and ethnic populations would help to account for, or even delineate, the effects of genetic ancestry.

Experiments in model organisms could be useful for investigating the mechanisms underlying phenotypic differences resulting from increased salivary amylase activity. At present, most studies assessing the effects (or potential causes) of varying *AMY1* CN have involved comparisons between mammalian species or human (modern, ancient, and archaic) populations (58, 59, 63); observational and clinical trials with human participants (Tables 1–3); and *in vitro* or mouse models inoculated with human microbiota from donors with varying *AMY1* CN (122, 140). Currently, there is at least one *Amy1* knockout mouse model (141). In an attempt to increase *Amy1* expression, two transgenic mouse lines with an insertion of the salivary amylase gene variant, *Amy-1.1*, from the YBR-Ki mouse strain were created (142). This resulted in increased amylase expression in the liver, but not the parotid gland, even though the inserted transgenes included promotors for expression in both the liver and the parotid gland. Several mouse lines with varying expression of salivary amylase would be a valuable tool. Scientists could control the amount and type of carbohydrate present in the mouse diet and study changes in body composition, insulin resistance, and the microbiota of the small and large intestine over time. They could also conduct glucose and meal tolerance tests during which they measure glucose, insulin, and incretin responses. These studies could provide insight into how *AMY1* CN contributes to glucose homeostasis and changes in adiposity.

There is potential to use *AMY1* CN as a risk factor for T2D and in combination with SAA and gut microbiome composition to custom tailor dietary recommendations. This would require additional dietary intervention studies in different populations. These studies should allow for the assessment of other candidate predictors to maximize relevance/utility for informing precision nutrition protocols. Additional factors, such as health status, sex, and age, should be assessed for their potential to interact with *AMY1* CN in shaping individual responses. Studies should include the assessment of incretin response to carbohydrates. These data could help clarify the underlying mechanisms by which salivary amylase impacts metabolic health. The efforts to do this are worthwhile because of the number of people who live with type 2 diabetes and obesity worldwide.

## 6 Conclusion

In this narrative review, we evaluated the evidence for associations between *AMY1* CN and metabolic health with a focus on adiposity, glucose homeostasis, and gut microbiome composition and function. The association between *AMY1* CN and adiposity is likely obfuscated by factors including other genes, carbohydrate intake, and other lifestyle factors. People with high *AMY1* CN tend to have a higher PPGR response to starchy foods than people with low *AMY1* CN. Counterintuitively, high *AMY1* CN appears to have a long-term protective effect on glucose homeostasis. But future studies, including observational and dietary interventions, are necessary for confirmation in different populations and to work out the contributing mechanisms—which likely include the gut microbiota.
